# Robust Myocardial Motion Tracking for Echocardiography: Variational Framework Integrating Local-to-Global Deformation

**DOI:** 10.1155/2013/974027

**Published:** 2013-03-11

**Authors:** Chi Young Ahn

**Affiliations:** Department of Computational Science and Engineering, Yonsei University, Seoul 120-749, Republic of Korea

## Abstract

This paper proposes a robust real-time myocardial border tracking algorithm for echocardiography. Commonly, after an initial contour of LV border is traced at one or two frames from the entire cardiac cycle, LV contour tracking is performed over the remaining frames. Among a variety of tracking techniques, optical flow method is the most widely used for motion estimation of moving objects. However, when echocardiography data is heavily corrupted in some local regions, the errors bring the tracking point out of the endocardial border, resulting in distorted LV contours. This shape distortion often occurs in practice since the data acquisition is affected by ultrasound artifacts, dropouts, or shadowing phenomena of cardiac walls. The proposed method is designed to deal with this shape distortion problem by integrating local optical flow motion and global deformation into a variational framework. The proposed descent method controls the individual tracking points to follow the local motions of a specific speckle pattern, while their overall motions are confined to the global motion constraint being approximately an affine transform of the initial tracking points. Many real experiments show that the proposed method achieves better overall performance than conventional methods.

## 1. Introduction

In company with the development of real-time three-dimensional echocardiography (RT3DE), the demands for automated analysis methods of left ventricle (LV) assessment such as ejection fraction, motion analysis, and strain analysis are rapidly increasing. Nevertheless, most of the analysis methods are still based on the measurements in a few two-dimensional (2D) slices, because they are available in clinical practice [1, 2]. In general, the quantitative assessment for heart function is performed by manually tracing endocardial border in some 2D slices of different view at frames (such as end-systole (ES) or end-diastole (ED) frames) selected from the entire cardiac cycle and automatically tracking the traced LV contour over the remaining frames [3, 4]. The motion tracking of LV is carried out by observing the speckle pattern associated with deforming tissue. Speckle pattern is an inherent appearance in ultrasound imaging and its local brightness reflects the local echogeneity of the underlying scatterers. Since it is a difficult task to automatically track the motion of endocardial border in ultrasound images due to ultrasound artifacts, dropouts or shadowing phenomena, low contrast, and so on, user intervention is somewhat required for stable and successful tracking of endocardial border.

In the last decades, there have been numerous studies for tracking of LV wall motion such as the tracking methods using deformable models [5–8], active shape models [9–11], and optical flow methods [2, 12–15]. Those methods have some limitations to practical application of endocardial border motion tracking. In deformable models, their methods are relatively time consuming due to iterative contour evolution with stopping criteria and often need preprocessing for speckle reduction before wall motion tracking. Active shape models are the statistical methods based on the dataset of trained images so that they require additional effort to train on many images. Both deformable models and active shape models provide the motion information of LV border and enable user to measure the volume inside LV, whereas they are somewhat inadequate for strain analysis related to the motion and deformation of heart, because they are not speckle tracking-based methods providing motion information of local region on the myocardium but shape-based tracking methods.

**Figure 1:**
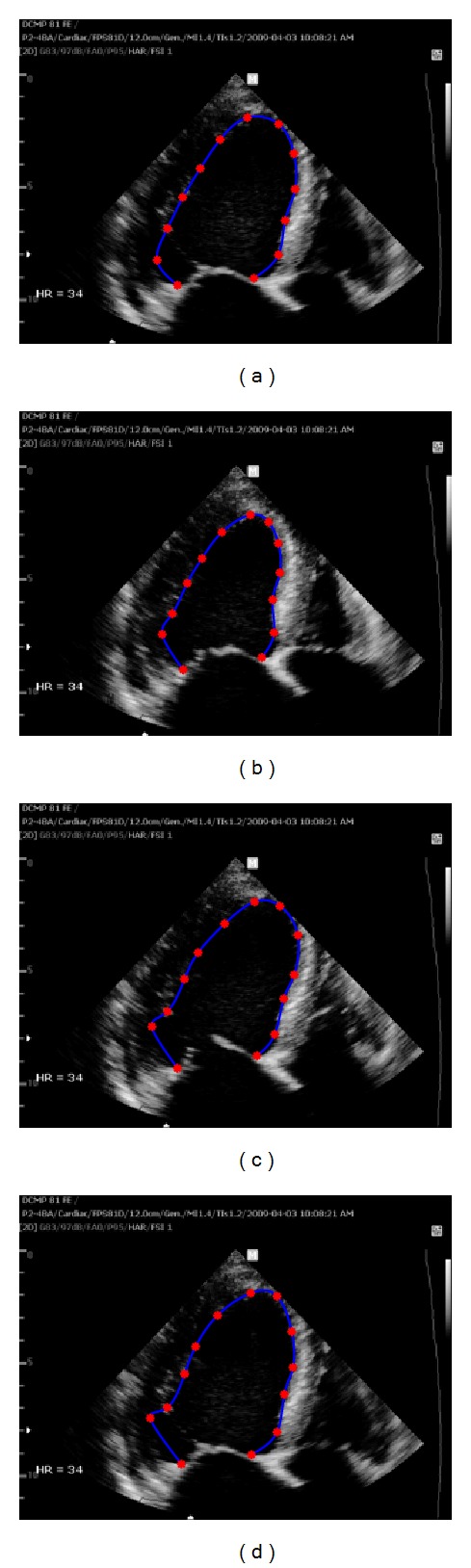
The estimation of endocardial border by the Lucas-Kanade optical flow method. Case  1: a tracking point getting out from the real LV shape distorts the whole shape near the border with weak edges; (a) initially traced endocardial border and its tracking points at an ED frame, (b) the tracked result at the ES frame, (c) at the frame between ES and ED, and (d) at the next ED frame.

**Figure 2:**
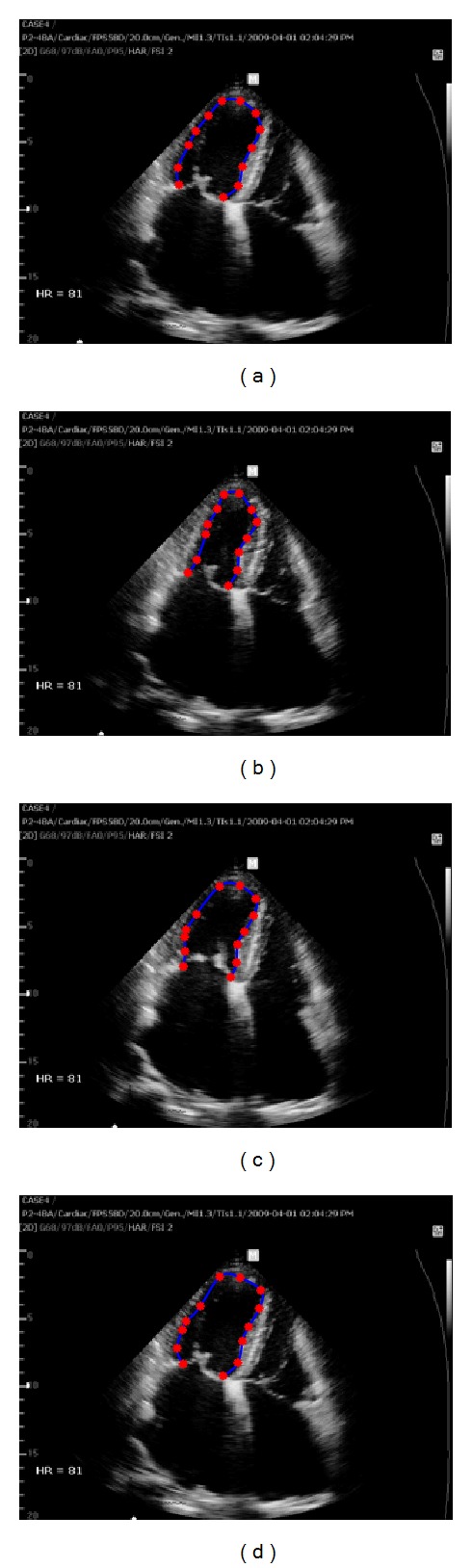
The estimation of endocardial border by the Lucas-Kanade optical flow method. Case  2: the tracked points are irregularly spaced by indistinguishable speckle patterns; (a) initially traced endocardial border and its tracking points at an ED frame, (b) the tracked result at the ES frame, (c) at the frame between ES and ED, and (d) at the next ED frame.

On the other hand, optical flow methods, which use the assumption that the intensity of a moving object is constant over time, provide the local motion information of myocardium. They are capable of measuring the LV volume as well as the myocardial wall motion analysis or strain analysis to detect LV abnormalities. After an initial contour of endocardial border is traced, each point on the contour tracks the specific intensity and speckle pattern in sequential images. However, it is problematic to track the endocardial border in ultrasound images with unclear speckle pattern or weak signals. In practical environment, there often exist some incorrectly tracked points due to ultrasound artifacts, dropouts, or shadowing phenomena of cardiac wall [16]. When edge dropout or indistinguishable speckle pattern is present in a local neighborhood of a tracking point, the errors bring the tracking point out of the endocardial border, resulting in distorted LV contours throughout the entire cardiac cycle as shown in Figure 1 or irregular distances between the tracked points in Figure 2. These distorted results affect LV volume measurement or strain analysis.

In order to cope with these problems, we develop a new optical flow method equipped with a global motion constraint that is designed to prevent each tracking point from getting out of the endocardial border. In the proposed model, the Lucas-Kanade (LK) optical flow method [17] and a global motion constraint being approximately an affine transformation of the initial tracking points are incorporated into a variational framework. So the individual tracking points follow speckle patterns (corresponding to each tracking point) and their overall motions are confined to the global motion constraint. The global motion constraint is based on the results [18, 19] that heart motion is regarded as the nonrigid motion by rotation, contraction/expansion, and shear. Typically, nonrigid motion consists of global deformation and local deformation. The global deformation is modeled by an affine transformation while the local deformation is described by a free-form deformation.

The proposed algorithm is capable of tracking LV border in real-time since its movement is directly computed from the difference between two sequential images via a simple matrix multiplication. For performance evaluation, we carry out various real experiments with Samsung Medison R&D Center (http://www.samsungmedison.com/). Numerous experiments show better performance of the proposed tracking methods compared to the conventional tracking methods. 

## 2. Methods

### 2.1. Conventional Optical Flow Tracking Methods

Let *I*(**r**, *t*) represent the intensity of echocardiography at the location **r** = (*x*, *y*) and the time *t*. Optical flow tracking methods are based on the assumption that the intensity of a moving object is constant over time, so that the noisy time-varying images *I*(**r**, *t*) approximately satisfy

(1) $$\begin{array}{c} u\left(r,t\right)\cdot \nabla I\left(r,t\right)+\frac{\partial }{\partial t}I\left(r,t\right)\approx 0, \end{array}$$

where **u**(**r**, *t*) is the velocity vector to be estimated. Based on (1), numerous approaches for estimating the velocity vector **u**(**r**, *t*) have been proposed and those were applied to LV border tracking in echocardiography [2, 13–15].

Horn and Schunk [20] proposed the optical flow technique incorporating the smoothness of the motion vector in the entire image as a global constraint. In their model, the velocity **u**(**r**, *t*) at each time *t* is determined by minimizing the energy functional:

(2) $$\begin{array}{c} E_{t}\left(u\right):=\int_{\Omega }{\left(u\left(r\right)\cdot \nabla I\left(r,t\right)+\frac{\partial }{\partial t}I\left(r,t\right)\right)}^{2}+\lambda {\left|\nabla u\left(r\right)\right|}^{2}dr, \end{array}$$

where *Ω* is the image domain and *λ* a regularization parameter which controls the balance between the optical flow term and the smoothness on **u**. The velocity **u**(**r**, *t*) at each time *t* can be computed by solving the corresponding Euler-Lagrange equation that is a reaction-diffusion equation. In [21], it has been observed that this global method with the global smoothness constraint is significantly more sensitive to noise than the local method used by Lucas and Kanade [17].

Lucas and Kanade [17] used the assumption of locally constant motion to compute the velocity **u**(**r**_0_, *t*) at a target location **r**_0_ = (*x*_0_, *y*_0_) and time *t* by forcing constant velocity in a local neighborhood of a point **r**_0_ = (*x*_0_, *y*_0_), denoted by *𝒩*(**r**_0_). Following Lucas and Kanade, Barron et al. [21] estimated the velocity **u**(**r**_0_, *t*) by minimizing the weighted least square criterion in the neighborhood *𝒩*(**r**_0_):

(3) u(r0,t)   :=arg min⁡u∫𝒩(r0)[w(r−r0)(u·∇I(r,t)+∂∂tI(r,t))2]dr,

where *w* is a weight function that enables to give more relevance to central terms rather than the ones in the periphery. Here, “arg min” stands for the argument of the minimum, that is, the vector **u** for which the right integral attains its minimum value. Since this method determines **u**(**r**_0_, *t*) at each location **r**_0_ by combining information from all pixels in the neighborhood of **r**_0_, it is reasonably robust against image noise. We used (3) as the Lucas-Kanade method, because this weighted window LK method is essentially same as the LK method. When the weight function *w* is uniform, the form is the same as the Lucas and Kanade one, in fact.

As we mentioned in Section 1, there often exist some incorrectly tracked points due to weak signal on cardiac wall since echocardiography data is acquired through transmitting and receiving ultrasound signals between the ribs, causing considerable shadowing of cardiac wall [16]. Due to these incorrectly tracked points, LK method may produce significantly distorted LV shape.

Recently, Sühling et al. [13] improved the weighted window LK method (3) by introducing a linear model for the velocity along the time direction, and the displacement **u**(**r**_0_, *t*) is obtained by evaluating **u** such that **u**, **b** ∈ ℝ^2^ and 2 × 2 matrix *A* minimize the following energy functional:

(4) Et(u,A,b)≔∫−∞∞∫ℝ2×[w(r−r0,s)((u+A(r−r0)+sb)·∇I(r,t+s)+∂∂tI(r,t+s))2]dr ds,

where *w* is the symmetric window function, which gives more weight to constraints at the center of the local spatiotemporal region than to those at the periphery. Since this method uses multiple frames centering around the time *t*, it is more robust than the LK method (3) using the single frame at *t*. However, the same problem of LV shape distortion as in LK method still remains.

Compared with the approaches based on the LK method, Duan et al. [15] used the region-based tracking method (also known as the block matching or pattern matching method) with the cross-correlation coefficients as a similarity measure. For given two consecutive images *I* at time *t* and *t* + Δ*t*, the velocity vector **u** = (*u*, *v*) for each pixel **r** = (*x*, *y*) ∈ *Ω* is estimated by maximizing the cross-correlation coefficients:

(5) u(r0,t) ≔  arg max⁡  u ×{∫𝒩(r0)[I(r,t)I(r+u,t+Δt)]dr∫𝒩(r0)[I(r,t)]2dr∫𝒩(r0)[I(r+u,t+Δt)]2dr}.

Instead of maximizing the cross-correlation coefficients, the velocity vector can be estimated by minimizing the sum-of-squared difference (SSD) [21] as follows:

(6) u(r0,t):=  arg min⁡u∫𝒩(r0) ×w(r−r0)[I(r,t)−I(r+u,t+Δt)]2dr.

The block matching method uses similarity measures that are less sensitive to noise, of fast motion, and of potential occlusions and discontinuities [15].

The above three local methods have drawback in dealing with the problem of the contour shape distortion in the presence of locally weak signal corrupted by rib shadowing and other factors. Hence, we need to develop a method alleviating shape distortion.

### 2.2. Proposed Method

 The proposed method uses an affine transformation to describe a global motion that is synthesized by integrating local deformations. We denote the endocardial border traced at initially selected frame (e.g., end-systole or end-diastole frame) by a parametric contour 𝒞* = {r*(*s*) = (x*(*s*), y*(*s*)) | 0 ≤ *s* ≤ 1} that can be identified as its *n* tracking points **r**_1_* = r*(*s*_1_),…, **r**_n_* = r*(*s*_*n*_). Here, 0 = *s*_1_ < *s*_2_ < ⋯<*s*_*n*_ = 1. Let *𝒞*(*t*) = {**r**(*s*, *t*) = (*x*(*s*, *t*), *y*(*s*, *t*)) | 0 ≤ *s* ≤ 1} be the contour deformed from *𝒞*(0) = 𝒞* at time *t*. The motion of the contour *𝒞*(*t*) will be determined by an appropriately chosen velocity **U**(*t*) indicating a time change of tracking points (**r**_1_(*t*),…, **r**_*n*_(*t*)):

(7) $$\begin{array}{c} U\left(t\right):=\left[\begin{array}{c} u_{1}\left(t\right)\\ \vdots \\ u_{n}\left(t\right) \end{array}\right]=\frac{d}{dt}\left[\begin{array}{c} r_{1}\left(t\right)\\ \vdots \\ r_{n}\left(t\right) \end{array}\right]\;\text{with}\left[\begin{array}{c} r_{1}\left(0\right)\\ \vdots \\ r_{n}\left(0\right) \end{array}\right]=\left[\begin{array}{c} r_{1}^{*}\\ \vdots \\ r_{n}^{*} \end{array}\right]. \end{array}$$

Here, we identify the contour *𝒞*(*t*) with tracking points (**r**_1_(*t*),…, **r**_*n*_(*t*)).

In our method, **U**(*t*) for each time *t* is a minimizer of the following energy functional reflecting local-to-global deformation:

(8) ℰt(U)≔12∑i=1n ×[∫𝒩(ri(t))w(r′−ri(t)){ui·∇I(r′,t)+∂∂tI(r′,t)}2dr′+λ|ri(t)+ui−[a1(U)a2(U)a3(U)a4(U)]ri∗−[a5(U)a6(U)]|2],

where *λ* is a nonnegative parameter, *w* is the weight function as used in the LK method, and the affine coefficients *a*_1_(**U**),…, *a*_6_(**U**) at time *t* are given by

(9) [a1(U)a3(U)a2(U)a4(U)a5(U)a6(U)]=(Φ(𝒞∗)TΦ(𝒞∗))−1Φ(𝒞∗)T×[(r1(t)+u1)T⋮(rn(t)+un)T],

where

(10) $$\begin{array}{c} \Phi \left({𝒞}^{*}\right):=\left[\begin{array}{cc} {r_{1}^{*}}^{T} & 1\\ \vdots & \vdots \\ {r_{n}^{*}}^{T} & 1 \end{array}\right]. \end{array}$$

The first term in (8) controls the individual tracking points to follow the local motions of a specific speckle pattern, while the second term controls their overall motions to be confined to the global motion constraint being approximately an affine transform of the initial tracking points.

The first term in (8) reflects the well-known LK optical flow (3) that probes local motions using blood-to-tissue intensity ratio.

The second term concerns a misfit between the estimated tracking points and their projection onto the space *𝒲*, the space of affine transforms of the initial tracking points, given by

(11) $$\begin{array}{c} 𝒲=\left\{\left[\begin{array}{cc} {r_{1}^{*}}^{T} & 1\\ \vdots & \vdots \\ {r_{n}^{*}}^{T} & 1 \end{array}\right]\left[\begin{array}{cc} a_{1} & a_{3}\\ a_{2} & a_{4}\\ a_{5} & a_{6} \end{array}\right]:\;a_{1},\ldots ,a_{6}\in \mathbb{R}\right\}\subset {\mathbb{R}}^{n\times 2}. \end{array}$$

To be precise, a careful computation yields

(12) the projection of    [(r1(t)+u1)T⋮(rn(t)+un)T]  onto  𝒲=Φ(𝒞∗)(Φ(𝒞∗)TΦ(𝒞∗))−1Φ(𝒞∗)T[(r1(t)+u1)T⋮(rn(t)+un)T]=[r1∗T1⋮⋮rn∗T1][a1(U)a3(U)a2(U)a4(U)a5(U)a6(U)].

Hence, the second term in (8) with the above identity reflects a global motion involving contraction, expansion, translation, and rotation.

To compute the minimizer **U** of the energy functional (8), we need to derive the Euler-Lagrange equation which can be obtained by taking partial derivative of *ℰ*_*t*_ with respect to each **u**_*j*_:

(13) 0=∂ℰt∂uj=∫𝒩(rj(t))w(r−rj(t))∇I(r,t)×{uj·∇I(r,t)+∂∂tI(r,t)}dr+λ{rj(t)+uj−∑i=1nd(i,j)(ri(t)+ui)}, for  j=1,…,n,

where *d*(*i*, *j*) is the (*i*, *j*)-component of the *n* × *n* matrix

(14) $$\begin{array}{c} 𝒫\left({𝒞}^{*}\right):=\Phi \left({𝒞}^{*}\right){\left(\Phi {\left({𝒞}^{*}\right)}^{T}\Phi \left({𝒞}^{*}\right)\right)}^{-1}\Phi {\left({𝒞}^{*}\right)}^{T}. \end{array}$$

The derivation of the Euler-Lagrange equation is given in the appendix.

For numerical algorithm, we replace the integral over *𝒩*(**r**_*j*_(*t*)) in (13) by summation over pixels around **r**_*j*_(*t*). Assuming that the neighborhood *𝒩*(**r**_*j*_(*t*)) consists of *m* pixels **r**_*j*1_,…, **r**_*jm*_, (13) becomes

(15) 0=AjTWjAjuj+AjTWjbj+λ×{rj(t)+uj−∑i=1nd(i,j)(ri(t)+ui)},

where *A*_*j*_ = [∇*I*(**r**_*j*1_, *t*),…, ∇*I*(**r**_*jm*_, *t*)]^*T*^, *W*_*j*_ = diag⁡(*w*(**r**_*j*1_ − **r**_*j*_(*t*)),…, *w*(**r**_*jm*_ − **r**_*j*_(*t*))), and **b**_*j*_ = [(∂/∂*t*)*I*(**r**_*j*1_, *t*),…, (∂/∂*t*)*I*(**r**_*jm*_, *t*)]^*T*^.

For notational simplicity, let the time *t* be fixed and let

(16) uj:=[ujvj],  rj(t)=[xjyj],[αjβjβjγj]:=AjTWjAj+λI,  [ξjηj]=AjTWjbj+λrj(t).

Then, the system (15) can also be represented by

(17) 0=αjuj+βjvj−λ∑i=1nd(i,j)ui+ξj−λ∑i=1nd(i,j)xi,0=βjuj+γjvj−λ∑i=1nd(i,j)vi+ηj−λ∑i=1nd(i,j)yi.

This can be concisely written by

(18) (Λ−λ𝒫(𝒞∗))U+BV=−Ξ+λ𝒫(𝒞∗)X,BU+(Γ−λ𝒫(𝒞∗))V=−Π+λ𝒫(𝒞∗)Y,

where Λ = diag⁡(*α*_1_,…, *α*_*n*_), *B* = diag⁡(*β*_1_,…, *β*_*n*_), Γ = diag⁡(*γ*_1_,…, *γ*_*n*_), *U* = [*u*_1_,…, *u*_*n*_]^*T*^, *V* = [*v*_1_,…, *v*_*n*_]^*T*^, *Ξ* = [*ξ*_1_,…, *ξ*_*n*_]^*T*^, Π = [*η*_1_,…, *η*_*n*_]^*T*^, *X* = [*x*_1_,…, *x*_*n*_]^*T*^, and *Y* = [*y*_1_,…, *y*_*n*_]^*T*^. Using the block matrix form, we can rewrite it as the system of linear equations:

(19) [(Λ−λ𝒫(𝒞∗))BB(Γ−λ𝒫(𝒞∗))][UV] =[−Ξ+λ𝒫(𝒞∗)X−Π+λ𝒫(𝒞∗)Y].

Therefore, we can directly compute the movement $U=\left[\begin{array}{cc} U & V \end{array}\right]$ of size *n* × 2 from the formula:

(20) [UV]=[(Λ−λ𝒫(𝒞∗))BB(Γ−λ𝒫(𝒞∗))]−1[−Ξ+λ𝒫(𝒞∗)X−Π+λ𝒫(𝒞∗)Y],

because the column vectors of the block matrix $\left[\begin{array}{cc} \left(\Lambda -\lambda 𝒫({𝒞}^{*})\right) & B\\ B & \left(\Gamma -\lambda 𝒫({𝒞}^{*})\right) \end{array}\right]$ of size 2*n* × 2*n* are linearly independent.

For the parameter *λ* = 0, the displacements **u**_*j*_  (*j* ∈ {1,…, *n*}) by (15) are exactly the same as those by the LK optical flow. However, (20) has a distinction to be capable of controlling the global shape in that the bigger the parameter *λ* is, the stronger the shape constraint is imposed. The LK optical flow performs a role as the local deformation subject to the global shape constraint, which is represented by the relationship of all *n* tracking points. Therefore, each point efficiently tracks maintaining the global deformation of initial LV contour.

### 2.3. Heuristic Choice of Parameter *λ*

For heuristic choice of parameter *λ*, we use various datasets of manually delineated LV borders by clinical experts. With manually defined data **r**_*j*_, 𝒞*, and  **U**(*t*) in a given image *I*, we define the parameter $\tilde{\lambda }$ as a function of quantity **r**_*j*_, 𝒞*, **U**(*t*), *I*, and time *t*:

(21) $$\begin{array}{c} \tilde{\lambda }:=\sqrt{\frac{\sum_{j=1}^{n}{||A_{j}^{T}W_{j}A_{j}u_{j}\left(t\right)+A_{j}^{T}W_{j}b_{j}||}_{2}^{2}}{\sum_{j=1}^{n}{||r_{j}\left(t\right)+u_{j}\left(t\right)-\sum_{i=1}^{n}d\left(i,j\right)\left(r_{i}\left(t\right)+u_{i}\left(t\right)\right)||}_{2}^{2}}}. \end{array}$$

We should note that if **u**_*j*_(*t*) satisfies (15) for all *t* and *j* = 1,…, *n*, then $\tilde{\lambda }=\lambda$, the constant independent of time *t*.

From numerous experiments, we observed that $\tilde{\lambda }$ tends to depend mainly on the contrast of the image *I*, and its dependency on time *t* is relatively small. We found a linear relationship between log⁡(*I*_tissue_/*I*_blood_) and $\tilde{\lambda }$, where *I*_tissue_/*I*_blood_ is an overall tissue/blood intensity ratio. 

**Figure 3:**
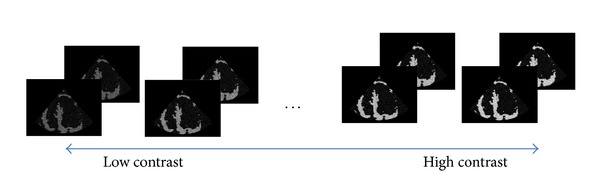
Image frames by varying tissue/blood intensity ratio. We use echographic texture modeling and heart motion modeling to generate image frames with various contrasts.

**Figure 4:**
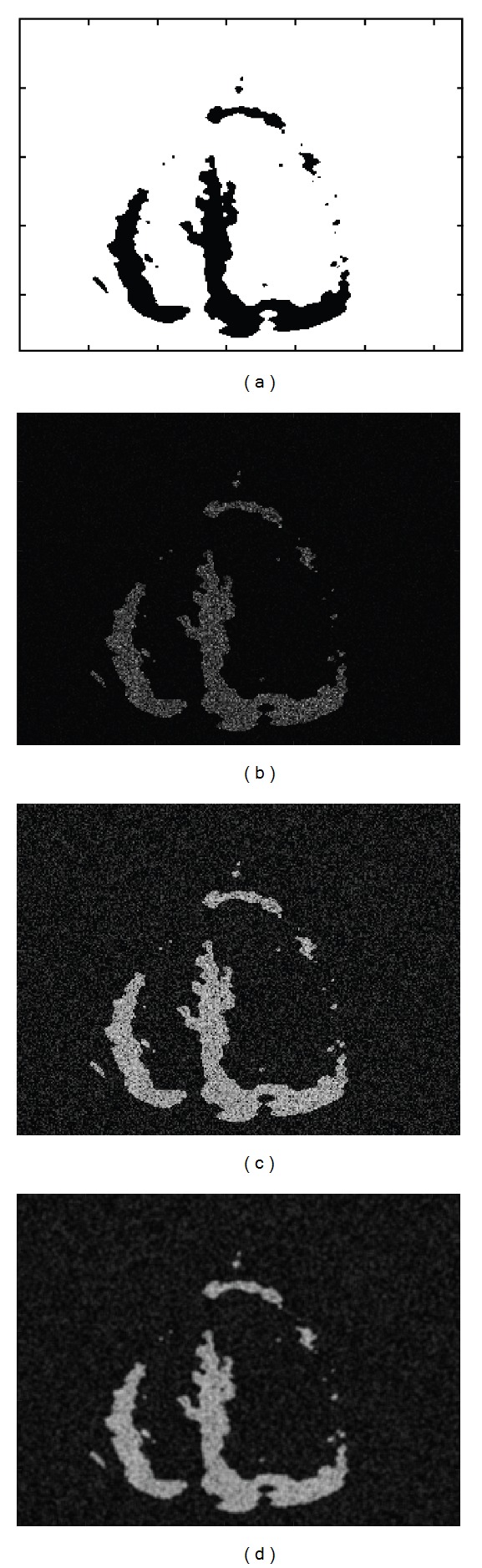
Synthetic images: (a) original LV template, (b) speckle image with Rayleigh distribution, (c) with Fisher-Tippet distribution, and (d) its smoothed image by a Gaussian filter.

To investigate behavior of the parameter $\tilde{\lambda }$, we generate synthetic speckle images consisting of tissue and blood regions and test them by changing conditions including tissue/blood contrast as shown in Figure 3. We use an apical long-axis view template shown in Figure 4(a). When the synthetic images are generated, it is assumed that speckle is fully developed so that the statistics of echo envelope follow the Rayleigh distribution ([22, 23]) and, by log-compression, the distribution of the intensities is changed into the Fisher-Tippett distribution ([24, 25])

(22) fσ(I) =2α1exp⁡{2α1(I−α2)−ln⁡(2σ2)−exp⁡      ×(2α1(I−α2)−ln⁡(2σ2))},

where *σ* is the distribution parameter represented in Rayleigh distribution and *α*_1_, *α*_2_ are the predetermined system parameters for log-compression of echo envelope. Finally, the synthetic images are smoothed by low-pass filter (in Figures 4(b), 4(c), and 4(d), resp.).

**Figure 5:**
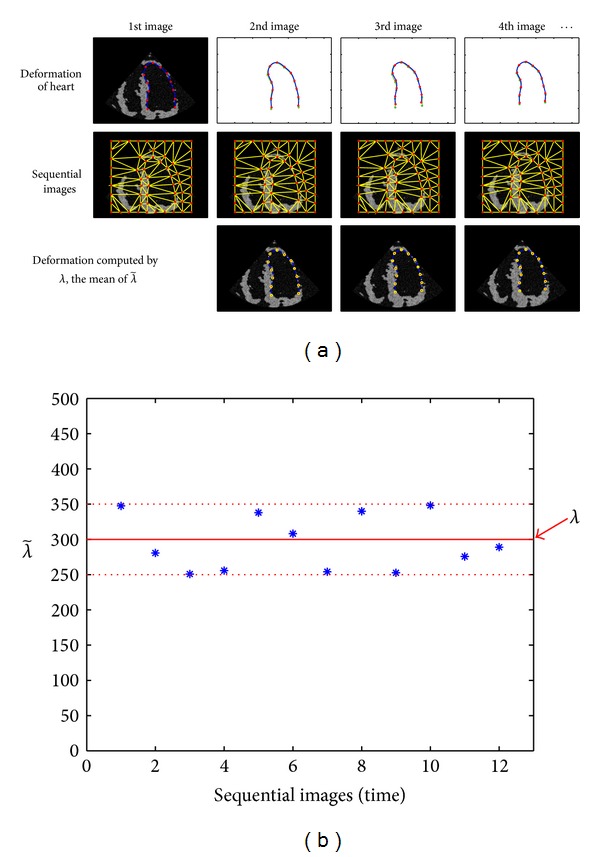
Result showing the independency of $\tilde{\lambda }$ with time *t*: (a) sequential synthetic images for myocardial motion. The first row shows the synthetic initial image and the tracking points representing the sequential motion of heart, the second row the sequential images corresponding to the motion of the tracking points, and the last row the tracking points (yellow “o” marks) and LV contour (yellow dotted line) by the displacements computed using *λ* = 300 and the mean value of $\tilde{\lambda }$ computed using the sequential images. (b) The change of $\tilde{\lambda }$ according to *t*. The $\tilde{\lambda }$ value varies within the range of [250, 350].

For modeling of heart motion, we simulate a heart with the nonrigid motion integrating global and local deformations.  Figure 5(a) illustrates the deformation of LV in four simulated images. LV contours are represented by 13 tracking points and a natural cubic spline connecting them, which are denoted by **r**_*i*_ at each time. Their *xy*-coordinates and displacements **u**_*i*_ from previous tracking points to next tracking points are listed in Table 1. For the sake of convenience in computation, it is assumed that the global deformation is modeled by an affine transformation of coefficients *a*_1_ = 0.92, *a*_2_ = −0.03, *a*_3_ = −0.01, *a*_4_ = 0.89, *a*_5_ = 20, and *a*_6_ = 6, which illustrate a contraction, and the local deformation is modeled by a free-form deformation of 0.1% variants with respect to the global deformation. In the first row of Figure 5(a), the blue solid lines and the red asterisks are showed as LV contour and tracking points by the defined heart motion, respectively. The green lines and asterisks mean LV contour at the previous frame. To generate the sequential images indicating the heart deformation, we also generate the tracking points of the epicardial contours so that the wall thickness between two contours is changed from 20 to 25 pixels in the sequential images. Using node points containing the endocardial and epicardial tracking points, the Delaunay triangulation meshes are generated and the sequential images are filled using linear spatial transformation from each mesh at previous image to the corresponding mesh at next image (second row).

We first test the dependency of $\tilde{\lambda }$ on the time *t*. For the given sequential synthetic images and tracking points at each time step, we compute $\tilde{\lambda }$ and plot the change of $\tilde{\lambda }$ with time *t*. The parameter $\tilde{\lambda }$ varies within the range of 250 to 350 as shown in Figure 5(b). Using *λ* = 300, the mean value of $\tilde{\lambda }$, we again compute (20) and get the displacements having the errors within 1 pixel compared to the reference displacements in Table 1(b). In this test, we use the 2-dimensional Gaussian function of variance *σ*_*x*_^2^ = *σ*_*y*_^2^ = 5^2^ (pixel size) for the weight function *w* over the square neighborhood with side length 21 pixels. From this test, we observe that the dependency of $\tilde{\lambda }$ on the time *t* is negligibly small.

**Figure 6:**
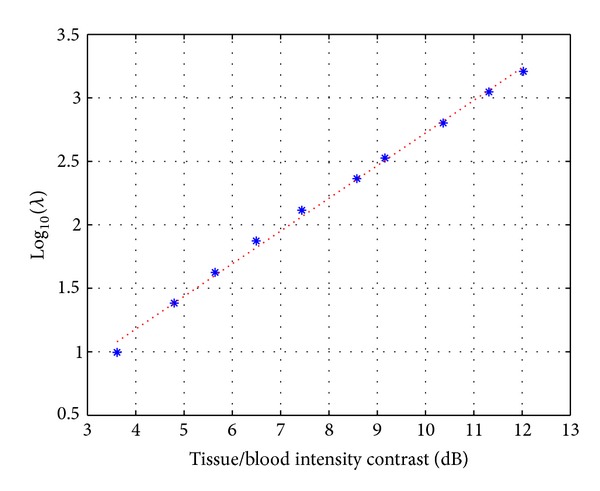
Graphs showing the relationship between $\tilde{\lambda }$ and the tissue/blood intensity contrast. The stars are the points obtained from the simulation; the straight line fits these points.

Next, we test the dependency of $\tilde{\lambda }$ on the tissue/blood intensity ratio. We generate the two consecutive images by varying the intensity of tissues as mentioned in Figure 3 and evaluate the change of $\tilde{\lambda }$ with respect to the tissue/blood intensity ratio.  Figure 6 shows that the relationship between the image intensity contrast and $\log_{10}\tilde{\lambda }$ is approximately linear. This linear relationship enables us to provide a way of choosing the parameter *λ* depending on the tissue/blood intensity ratio effectively. 

## 3. Experimental Results

We test the proposed algorithm in clinical setting using many real data. We compare the performance of the proposed algorithm with some widely used tracking algorithms including the block matching tracking methods using sum-of-squared difference (SSD) and cross-correlation coefficient, and the LK optical flow. For experiments, we use the 35 cases of 240 × 320 size 2D echocardiography data acquired using a Samsung Medison V10 ultrasound system (Seoul, Republic of Korea) and a phased array transducer P2-4BA (2–4 MHz). We use 19 tracking points to track the endocardial border and make the LV contour connecting the points using the natural cubic spline. All the experiments were conducted using MATLAB 7.5 and laptop computer (Inter processor U7300 at 1.3 GHz and 1 GB RAM), and the computational time was about 40 milliseconds at each frame.

### 3.1. Assessment of LV Border Tracing

A quantitative evaluation on the performance of the proposed tracking algorithm is done on real 2D image sequences. For computation of **u** · ∇*I* + (∂/∂*t*)*I*, we use the standard finite difference method. We use the Hausdorff distance *ε*_*H*_ [26, 27] to compare the automated LV contours produced by algorithms with manually traced contours by a clinical expert. Here, the Hausdorff distance between the contour *𝒞*_1_ and *𝒞*_2_ is given by

(23) εH(𝒞1,𝒞2)=max⁡{sup⁡r1∈C1(min⁡r2∈C2||r1−r2||),sup⁡r2∈C2(min⁡r1∈C1||r1−r2||)}.

**Figure 7:**
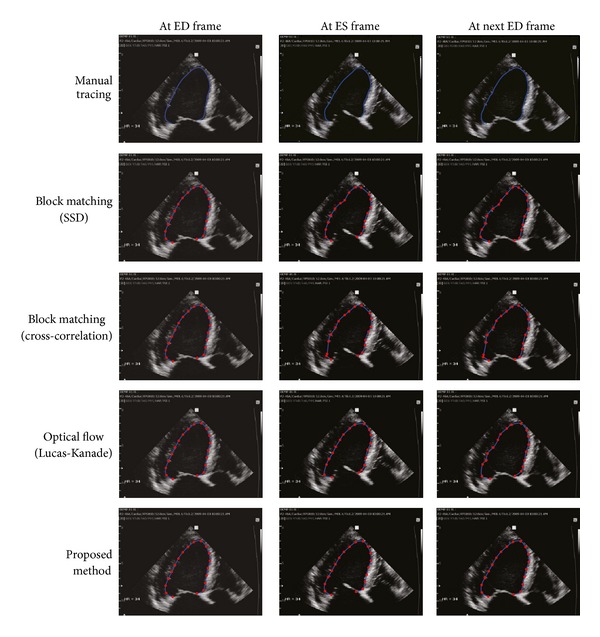
Case I: real images with weak signals in endocardial border. The second and third rows are the results by region-based tracking methods using sum-of-squared difference (SSD) and cross-correlation, respectively. The fourth row is the result by the LK optical flow and the final row is the result by the proposed method (*λ* = 120).

**Figure 8:**
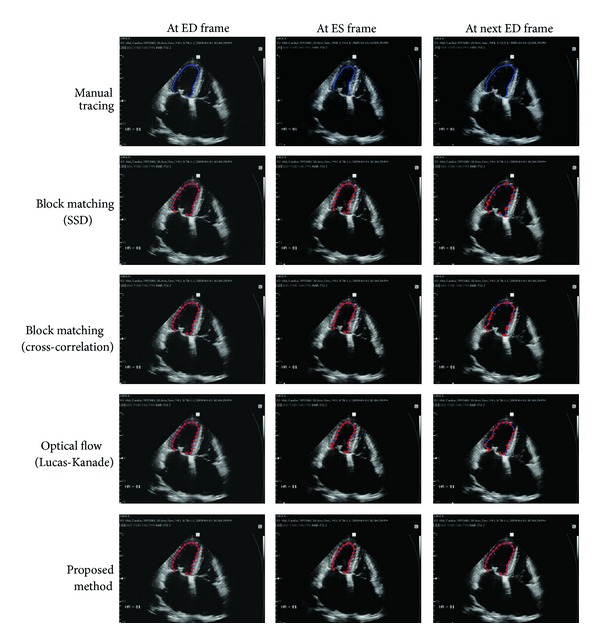
Case II: real images with indistinguishable speckle patterns in endocardial border. The second and third rows are the results by region-based tracking methods using sum-of-squared difference (SSD) and cross-correlation, respectively. The fourth row is the result by the LK optical flow and the fifth row by the proposed method (*λ* = 100).

For two representative cases among the 35 cases of 2D echocardiography data, the LV tracking results of the proposed method and the conventional methods are shown in Figures 7 and 8, respectively. For the sake of a name, we call them Cases I and II.

In Figure 7, the first row is manually traced LV contours by a clinical expert for images at ED, ES, and ED frames in the entire cycle. The next two rows are results by two block matching tracking methods using the different similarity measures of sum-of-squared difference (SSD) and cross-correlation. The fourth and fifth rows are obtained by the LK optical flow and the proposed method, respectively. The three conventional methods produce distorted LV contours due to a few incorrect tracking points alienated from the real LV border. On the other hand, the proposed method successfully follows local speckle patterns without distorting the whole LV shape.

For initial 10 sequential images, we compute $\tilde{\lambda }$ by manually identifying each tracking point to the corresponding position on each image. From the computed parameters $\tilde{\lambda }$, *λ* is set to 120 according to the *λ*-choice method described in Section 2.3.

In Figure 8, we test for real images having indistinguishable speckle patterns near endocardial border. Due to the presence of indistinguishable speckle patterns, the three conventional methods produce irregular distribution of tracking points as shown in second, third, and fourth rows in Figure 8. The proposed method keeps regular distribution of tracking points and successfully track local speckle patterns. For Case II, *λ* is set to 100.

**Figure 9:**
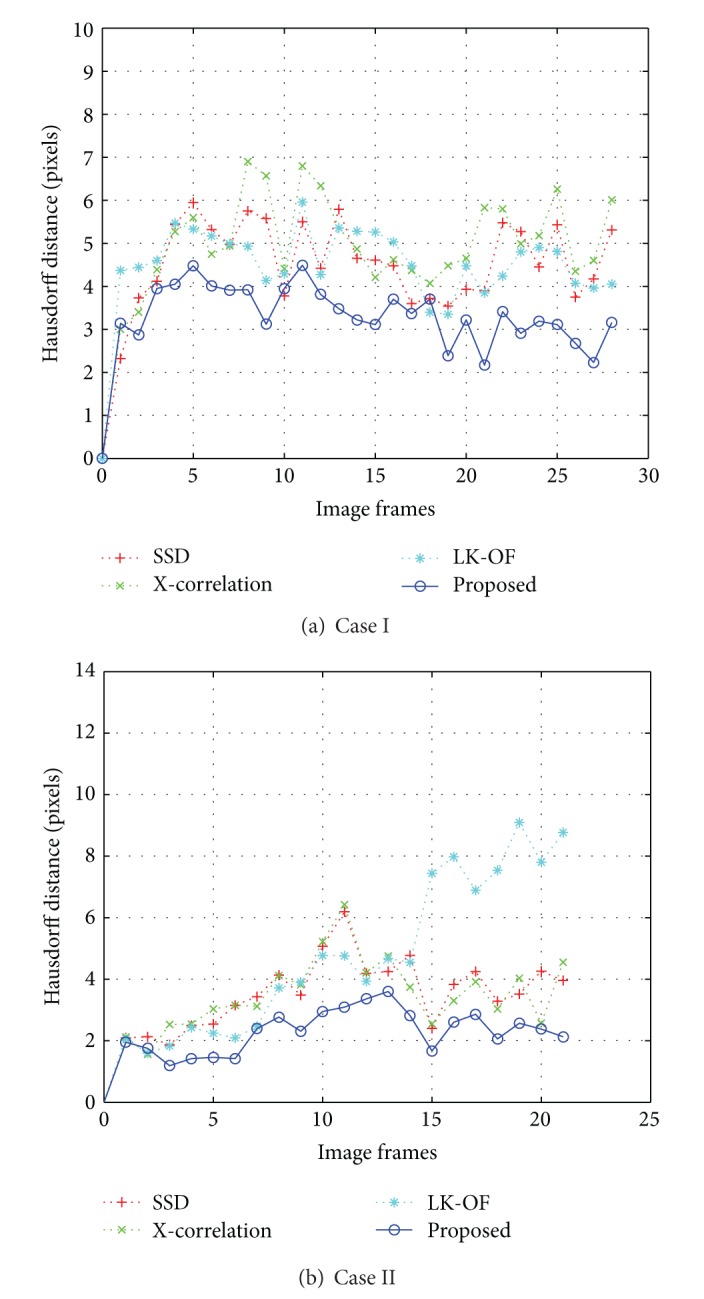
Comparison results of LV contours using the Hausdorff distance *ε*_*H*_ for the entire images of (a) Case I and (b) Case II, which are shown in Figures 7 and 8, respectively.

Figure 9 shows the comparison results of four different methods using Hausdorff distance between contours drawn manually and contours generated automatically for the entire cycle from an ED frame to the next ED frame. The proposed method provides the smallest errors in final tracking results of both Cases I and II.

### 3.2. Assessment of Individual Tracking Point Errors

For performance evaluation of the proposed algorithm, we propose an additional assessment regarding the repeatability of local point along the forward and backward entire cardiac cycle. Let {**r**_1_^initial^,…, **r**_*n*_^initial^} be the set of initial tracking points on a manually delineated contour (see the images of the left column in Figure 7). Let *t*_*R*_ be a time interval of a one cycle image between ED frame and the next ED frame. Using one cycle image *I*(**r**, *t*), 0 ≤ *t* ≤ *t*_*R*_, we generate a forward-backward image defined by

(24) $$\begin{array}{c} \tilde{I}\left(r,t\right)=\{\begin{array}{cc} I\left(r,t\right) & \text{if}\;0\leq t\leq t_{R}\\ I\left(r,2t_{R}-t\right) & \text{if}\;t_{R}\leq t\leq 2t_{R}. \end{array} \end{array}$$

Using this forward-backward image $\tilde{I}\left(r,t\right),\;0\leq t\leq 2t_{R}$, we apply an automated tracking algorithm to get the returning tracking position **r**_*j*_^returning  ^ at time *t* = 2*t*_*R*_. The local tracking point assessment is obtained by estimating the distance between the initial position **r**_*j*_^initial  ^and the corresponding returning position **r**_*j*_^returning^:

(25)   Forward-backward  point  tracking  error  (FBTE)    =1n∑i=1n|riinitial−rireturning|2.

For the previous two representative cases, Cases I and II, Table 2 shows the comparison results of the proposed method with the conventional methods using the FBTE.

Table 3 shows the mean and standard deviation of the forward-backward point tracking errors of the results obtained by the three conventional tracking methods and the proposed method. Tables 2 and 3 reveal that the proposed method provides improved performance compared with the conventional tracking methods.

## 4. Discussion and Conclusion

The proposed method controls the individual tracking points following optical flow by confining their overall motions by penalizing the misfit between the estimated tracking points and their projection onto the affine transform space *𝒲* in (11) of the initial tracking points.

We have experimentally demonstrated that the proposed method is capable of performing robust real-time LV border tracking even in the presence of indistinguishable portions of the LV walls in echocardiography data. In practice, echocardiography data often contains edge dropout or indistinguishable speckle patterns in a local neighborhood of a tracking point which may bring the tracking point out of the endocardial border, resulting in distorted LV contours. The proposed method effectively deals with these problems by taking advantage of an LV shape space describing a global motion that is synthesized by integrating local deformations governed by the LK optical flow model. Various experiments show that the proposed method achieves better overall performance than the widely used conventional methods including the block matching tracking methods using sum-of-squared difference (SSD) and cross-correlation, and the LK optical flow.

The proposed method performs the LV border tracking by directly computing the displacements between two sequential images via a simple matrix multiplication. The computational time is affected by the size of the matrix, depending on the number of tracking points.

We also proposed a new performance evaluation method for LV tracking that is based on the forward-backward tracking error estimation as shown in Section 3.2. The conventional evaluation of global tracking performance using the delineated LV contours has some limitations in estimating errors of individual tracking points; in the case when tracking points erroneously move along LV border, the LV contour connecting the tracking points cannot reveal those individual tracking errors. The forward-backward point tracking error estimation provides a better local tracking performance assessment in the whole cycle.

The proposed technique can be extended to three dimensions by using 3D affine transformation as a global deformation. 

## Figures and Tables

**Table:** (a) Tracking points

*i*	**r**_*i*_ at 1st *I*	**r**_*i*_ at 2nd *I*	**r**_*i*_ at 3rd *I*	**r**_*i*_ at 4th *I*
1	(152, 201)	(153, 196)	(154, 187)	(154, 183)
2	(151, 176)	(152, 171)	(153, 165)	(154, 161)
3	(155, 151)	(156, 147)	(157, 142)	(158, 139)
4	(152, 128)	(153, 125)	(155, 120)	(156, 118)
5	(142, 105)	(144, 103)	(146, 100)	(147, 98)
6	(147, 82)	(149, 80)	(152, 78)	(153, 77)
7	(167, 71)	(169, 70)	(171, 69)	(172, 68)
8	(188, 84)	(189, 82)	(190, 80)	(191, 79)
9	(207, 101)	(207, 99)	(207, 96)	(207, 94)
10	(217, 124)	(217, 121)	(216, 116)	(216, 114)
11	(224, 147)	(223, 143)	(222, 138)	(221, 135)
12	(229, 172)	(228, 167)	(227, 160)	(226, 156)
13	(229, 196)	(228, 191)	(226, 182)	(225, 178)

**Table:** (b) Displacements

*i*	**u**_*i*_ at 1st *I*	**u**_*i*_ at 2nd *I*	**u**_*i*_ at 3rd *I*
1	(1, −5)	(1, −9)	(0, −4)
2	(1, −5)	(1, −6)	(1, −4)
3	(1, −4)	(1, −5)	(1, −3)
4	(1, −3)	(2, −5)	(1, −2)
5	(2, −2)	(2, −3)	(1, −2)
6	(2, −2)	(3, −2)	(1, −1)
7	(2, −1)	(2, −1)	(1, −1)
8	(1, −2)	(1, −2)	(1, −1)
9	(0, −2)	(0, −3)	(0, −2)
10	(0, −3)	(−1, −5)	(0, −2)
11	(−1, −4)	(−1, −5)	(−1, −3)
12	(−1, −5)	(−1, −7)	(−1, −4)
13	(−1, −5)	(−2, −9)	(−1, −4)

**Table 2:** The comparison results of the proposed method with the conventional methods using FBTE, for Case I and Case II (in pixels).

Method	Case I	Case II
Block matching (SSD)	3.6992	4.6566
Block matching (cross-correlation)	2.3396	5.5866
Optical flow (LK)	2.6326	2.1521
Proposed method	0.4052	0.7930

**Table 3:** The comparison results of the tracking algorithms for the total experimental dataset of 35 cases. The errors are measured using the FBTE (in pixels).

Method	Mean of errors	Standard deviation of errors
Block matching (SSD)	4.1936	2.4456
Block matching (cross-correlation)	4.4173	2.5684
Optical flow (LK)	3.0685	1.2997
Proposed method	0.6344	0.2884

## Appendix

### A. Derivation of the Euler-Lagrange Equation (13)

In this appendix, we derive the Euler-Lagrange equation (13) from (8). From (9), we have

(A.1) $$\begin{array}{c} \left[\begin{array}{c} \frac{\partial a_{1}}{\partial u_{j}}\\ \frac{\partial a_{2}}{\partial u_{j}}\\ \frac{\partial a_{5}}{\partial u_{j}} \end{array}\right]={\left(\Phi {\left({𝒞}^{*}\right)}^{T}\Phi \left({𝒞}^{*}\right)\right)}^{-1}\left[\begin{array}{c} x_{j}^{*}\\ y_{j}^{*}\\ 1 \end{array}\right],\\ \left[\begin{array}{c} \frac{\partial a_{3}}{\partial u_{j}}\\ \frac{\partial a_{4}}{\partial u_{j}}\\ \frac{\partial a_{6}}{\partial u_{j}} \end{array}\right]=\left[\begin{array}{c} 0\\ 0\\ 0 \end{array}\right],\;\;\left[\begin{array}{c} \frac{\partial a_{1}}{\partial v_{j}}\\ \frac{\partial a_{2}}{\partial v_{j}}\\ \frac{\partial a_{5}}{\partial v_{j}} \end{array}\right]=\left[\begin{array}{c} 0\\ 0\\ 0 \end{array}\right],\\ \left[\begin{array}{c} \frac{\partial a_{3}}{\partial v_{j}}\\ \frac{\partial a_{4}}{\partial v_{j}}\\ \frac{\partial a_{6}}{\partial v_{j}} \end{array}\right]={\left(\Phi {\left({𝒞}^{*}\right)}^{T}\Phi \left({𝒞}^{*}\right)\right)}^{-1}\left[\begin{array}{c} x_{j}^{*}\\ y_{j}^{*}\\ 1 \end{array}\right], \end{array}$$

for *j* = 1,2,…, *n*. From the first and fourth identities of (A.1), we have

(A.2) d(i,j)=∂a1∂ujxi∗+∂a2∂ujyi∗+∂a5∂uj=[xi∗yi∗1][∂a1∂uj∂a2∂uj∂a5∂uj]=[xi∗yi∗1](Φ(𝒞∗)TΦ(𝒞∗))−1[xj∗yj∗1]=[xi∗yi∗1][∂a3∂vj∂a4∂vj∂a6∂vj]=∂a3∂vjxi∗+∂a4∂vjyi∗+∂a6∂vj.

Using (A.1) and (A.2), the partial derivatives ∂*ℰ*_*t*_/∂*u*_*j*_ and ∂*ℰ*_*t*_/∂*v*_*j*_ of *ℰ*_*t*_ with respect to **u**_*j*_ = (*u*_*j*_, *v*_*j*_) are computed by

(A.3) ∂ℰt∂uj=∫𝒩(rj(t))w(r′−rj(t)) ×{uj·∇I(r′,t)+∂∂tI(r′,t)}∂∂xI(r′,t)dr′ +λ[{xj+uj−(a1xj∗+a2yj∗+a5)}−∑i=1n{xi+ui−(a1xi∗+a2yi∗+a5)}(∂a1∂ujxi∗+∂a2∂ujyi∗+∂a5∂uj)]=∫𝒩(rj(t))w(r′−rj(t)) ×{uj·∇I(r′,t)+∂∂tI(r′,t)}∂∂xI(r′,t)dr′ +λ[{xj+uj−(a1xj∗+a2yj∗+a5)}   −∑i=1nd(i,j){xi+ui−(a1xi∗+a2yi∗+a5)}],

(A.4) ∂ℰt∂vj=∫𝒩(rj(t))w(r′−rj(t)) ×{uj·∇I(r′,t)+∂∂tI(r′,t)}∂∂yI(r′,t)dr′ +λ[{yj+vj−(a3xj∗+a4yj∗+a6)}−∑i=1n{yi+vi−(a3xi∗+a4yi∗+a6)}(∂a3∂vjxi∗+∂a4∂vjyi∗+∂a6∂vj)]=∫𝒩(rj(t))w(r′−rj(t)) ×{uj·∇I(r′,t)+∂∂tI(r′,t)}∂∂yI(r′,t)dr′ +λ[{yj+vj−(a3xj∗+a4yj∗+a6)}   −∑i=1nd(i,j){yi+vi−(a3xi∗+a4yi∗+a6)}].

Simple arrangement of the above identities leads to the Euler-Lagrange equation:

(A.5) 0=∂ℰt∂uj=∫𝒩(rj(t))w(r−rj(t))∇I(r,t)×{uj·∇I(r,t)+∂∂tI(r,t)}dr+λ{rj(t)+uj−gj(U)−∑i=1nd(i,j)(ri(t)+ui−gi(U))},

where $g_{i}(U)=\left[\begin{array}{cc} a_{1}(U) & a_{2}(U)\\ a_{3}(U) & a_{4}(U) \end{array}\right]r_{i}^{*}+\left[\begin{array}{c} a_{5}(U)\\ a_{6}(U) \end{array}\right]$. The Euler-Lagrange equation (13) can be obtained by a careful rearrangement of (13) using the identity

(A.6) $$\begin{array}{c} g_{j}\left(U\right)=\sum_{i=1}^{n}d\left(i,j\right)g_{i}\left(U\right). \end{array}$$

Hence, it remains to prove the above identity. It suffices to prove

(A.7) $$\begin{array}{c} \sum_{i=1}^{n}x_{i}^{*}d\left(i,j\right)=x_{j}^{*},\;\;\sum_{i=1}^{n}y_{i}^{*}d\left(i,j\right)=y_{j}^{*},\;\;\sum_{i=1}^{n}d\left(i,j\right)=1, \end{array}$$

for *j* = 1,2,…, *n*. To compute ∑_*i*=1_^*n*^ *x*_*i*_**d*(*i*, *j*) simply, denote

(A.8) $$\begin{array}{c} \left[\begin{array}{c} \phi_{j}\\ \varphi_{j}\\ \psi_{j} \end{array}\right]:={\left[\begin{array}{ccc} \sum_{k=1}^{n}x_{k}^{*2} & \sum_{k=1}^{n}x_{k}^{*}y_{k}^{*} & \sum_{k=1}^{n}x_{k}^{*}\\ \sum_{k=1}^{n}x_{k}^{*}y_{k}^{*} & \sum_{k=1}^{n}y_{k}^{*2} & \sum_{k=1}^{n}y_{k}^{*}\\ \sum_{k=1}^{n}x_{k}^{*} & \sum_{k=1}^{n}y_{k}^{*} & n \end{array}\right]}^{-1}\left[\begin{array}{c} x_{j}^{*}\\ y_{j}^{*}\\ 1 \end{array}\right]. \end{array}$$

Then ∑_*i*=1_^*n*^ *x*_*i*_**d*(*i*, *j*) can be simplified by

(A.9) ∑i=1nxi∗d(i,j) =∑i=1nxi∗[xi∗yi∗1] ×([x1∗y1∗1⋮xn∗yn∗1]T[x1∗y1∗1⋮xn∗yn∗1])−1[xj∗yj∗1] =[∑i=1nxi∗2∑i=1nxi∗yi∗∑i=1nxi∗] ×[∑k=1nxk∗2∑k=1nxk∗yk∗∑k=1nxk∗∑k=1nxk∗yk∗∑k=1nyk∗2∑k=1nyk∗∑k=1nxk∗∑k=1nyk∗n]−1[xj∗yj∗1] =[∑i=1nxi∗2∑i=1nxi∗yi∗∑i=1nxi∗][ϕjφjψj] =ϕj∑i=1nxi∗2+φj∑i=1nxi∗yi∗+ψj∑i=1nxi∗ =xj∗.

This completes the first identity of (A.7). Similarly, we can get the remaining two identities of (A.7).
